# Temperature Responsive PBT Bicomponent Fibers for Dynamic Thermal Insulation

**DOI:** 10.3390/polym14142757

**Published:** 2022-07-06

**Authors:** Ninad Khadse, Rebecca Ruckdashel, Shnaidie Macajoux, Hongwei Sun, Jay Hoon Park

**Affiliations:** 1 Department of Plastics Engineering, University of Massachusetts Lowell, Lowell, MA 01854, USA; ninad_khadse@student.uml.edu (N.K.); rebecca_ruckdashel@uml.edu (R.R.); shnaidie_macajoux@student.uml.edu (S.M.); 2 Department of Mechanical and Industrial Engineering, Northeastern University, Boston, MA 02115, USA; ho.sun@northeastern.edu

**Keywords:** thermoresponsive, bicomponent, melt spinning, thermal insulation

## Abstract

Thermoresponsive self-crimping polybutylene terephtlate (PBT)-based bicomponent fibers were fabricated by melt-spinning to serve as primary constituents for textiles, such as nonwoven battings, for an adaptive single insulting layer. Due to the intrinsically mismatching modulus and coefficient of thermal expansion (CTE), the fibers curl or straighten with temperature, similar to the concept of Timoshenko’s bimetallic strip. Maximizing the curvature is driven by an optimum of fiber diameter, overall CTE, and fiber moduli, which are all affected by drawing ratio and, consequently, fiber’s microstructure. A draw ratio of 2.33 yielded the best combination of mechanical and thermal properties; it was observed that increasing the draw ratio does not necessarily increase the self-crimping behavior. Tests performed on non-woven battings of these fibers exhibited comparable thermoreponsive behaviors to polypropylene-based thermoresponsive fibers from previous studies in the −20 °C to 20 °C temperature range, which has potential for wearable insulations for both commercial and defense sectors alike.

## 1. Introduction

Polybutylene terephthalate (PBT), along with polyethylene terephthalate (PET), belongs to the widely commercialized family of polyalkylene terephthalate. Although both PBT and PET are semi-crystalline, the distinct molecular structures give rise to faster crystallization shown for PBT which has popularized it for injection molding where shorter cycle times are essential [1]. Both PBT and/or PET could be spun into continuous filaments then subsequently made into textiles; as such, mechanical properties of textiles that can withstand strains imposed by wearing are important, while other factors, such as comfort related to thermal and moisture management, should be considered.

Commercial thermal comfort garments are based on resistive heating, e.g., an electrical blanket or Olympic team jackets [2], yet these garments have increased fire risk and require battery power. Alternatively, thermal comfort is enhanced by: (1) storage/release of latent heat with a phase change material coating [3,4], (2) stimuli-responsive fiber or fabric like shape memory materials [5], or (3) changing water vapor permeability with a stimuli-responsive hydrogel like chitosan-poly(NIPAAm) [6,7]. However, these approaches fail to meet requirements due to limited substrates with functional groups for grafting [6], susceptibility of coatings or electronics to damage during washing, or leakage and breakage of phase change materials.

A better solution can be found in nature: sheep’s wool. Wool’s cross section has an asymmetric structure due to protein expression which causes the fiber to crimp [8]. Crimping leads to irregularity in packing which traps air to insulate the heat to the wearer. Only fibers with “well-separated phases” along the fiber axis, e.g., side-by-side, core-shell, or bead on string, can exhibit “thermoresponsive behavior and reversible actuation” [9]. Carefully controlled asymmetric cooling can create “differential characteristics,” although the effect is small [10]. These intrinsically stimuli-responsive materials are found in solid-state polymers or composites therein [11]; such functionalities give rise to what is referred to as inherently “smart” fibers and/or textiles which do not lend itself to external electronic component [12].

One may mimic such smart thermal responsive behavior observed in nature by synthetic means of bicomponent melt spinning. Bicomponent fibers are a class of multi-component fibers where the two components can be arranged in geometrical configurations, e.g., side-by-side, core-sheath, islands-in-sea, or segmented-pie. For instance, electrospinning incompatible PNIPAAm and P(MMA-co-BMA) creates a two-phase structure which, depending on solvent, either responds to temperature with reversible buckling (transition around LCST ~27 °C) or responds to moisture with porosity variation (lower porosity in wetter climate) [9]. Asymmetric hydrophilicity carbon nanotube coated triacetate-cellulose bimorph fiber yarns, responds to temperature and humidity with an expansion/contraction for variable infrared emissivity and air permeability [13].

Side-by-side bicomponent melt coextrusion of fibers with different coefficient of thermal expansion (CTE) can yield thermoresponsive fibers. The fiber curvature with respect to temperature change for a bicomponent fiber was predicted between 25 to 80 vol% of high-CTE component depending on moduli ratio [14,15]; syndiotactic PP (slow crystallization/low modulus—high CTE) and isotactic PP (high crystallinity/high modulus—low CTE) bicomponent fiber was one such example. Based on Timoshenko’s beam bending theory [14], Equation (1) was derived for side-by-side bicomponent fiber curvature change which considers the coefficient of thermal expansion and the young’s modulus which, for polymers can vary by three orders of magnitude based on their glass transition temperature [15].

(1) $$C=\frac{1}{\rho }=\frac{\frac{\pi }{2}\left(\alpha_{2}-\alpha_{1}\right)\left(T-T_{0}\right)}{h\;\left[\left(\frac{\pi^{2}}{8}-\frac{8}{9}\right)+\left(\frac{\pi^{2}}{16}-\frac{4}{9}\right)n+\left(\frac{\pi^{2}}{16}-\frac{4}{9}\right)\frac{1}{n}\right]}$$

In Equation (1), for a fiber with a circular cross-section with each polymer component occupying a semicircle with a straight interface, *C* is the fiber curvature, *ρ* is the radius of curvature of the fiber, *α* is the coefficient of thermal expansion (CTE), *T* is the temperature, *T*_0_ is the reference temperature prior to curvature motion of the fiber, *n* is the ratio of Young’s moduli of the two components, and *h* is the height or diameter of the fiber. According to this equation, the curvature response is linear with CTE and temperature change. Since the height term is in the denominator, fibers with smaller diameters yield a greater curvature change response. Past studies have reported bi- and tri-component fibers made of isotactic and syndiotactic polypropylene which show up to 1.5% thickness change per °C [15,16].

Asymmetric side-by-side fibers with differential thermal elongation in pile fabrics curve when heated so that the fabric becomes thinner, more compact and less insulating [17]. However, components in bicomponent fibers may pull apart during repeated cycling due to weak interfacial bonding [15]. As such, consideration of miscibility between two components is crucial; this aspect depends on inherent miscibility of the materials and/or be enhanced with a compatibilizer [6] or an adhesive.

In this work, side-by-side bi-component thermoresponsive fiber with PBT-based polymers were produced; these materials demonstrate comparable radius of curvature change due to a CTE mismatch as previously reported PP-based smart fibers [14,15]. One advantage over the previous study [16] is that the miscibility of the two PBT components allows for fabrication without an adhesive or a compatibilizer. Moreover, the presence of a compatibilizer layer affects the maximum crimp potential of the fiber since the component fraction is not optimum due to the middle layer and modulus mismatch at each interface is reduced. The PBT resins used in the current study, Hytrel and Crastin, are readily available commercial resins, while syndiotactic PP used in the previous study [16] is no longer commercially available due to its low utility. The effect of CTE mismatch and draw ratio of the fiber was studied for single fibers in this study to quantify the expected self-crimping response as a function of temperature. The fibers were then made into textiles as a nonwoven batting to demonstrate the self-crimping thermoresponsive behavior and its implication for enhanced thermal resistance from inclement weather.

## 2. Materials and Methods

### 2.1. Materials

Polybutylene terephthalate (Crastin 6130) and a polyester thermoplastic elastomer (Hytrel 5526) obtained from DuPont de Nemours, Inc (Wilmington, DE, USA) were used to melt-spin side-by-side bicomponent fibers. Hytrel is a block co-polymer consisting of a crystallizable PBT hard segment and a soft segment consisting of poly(tetramethylene ether) glycol terephthalate which allows it to act as an elastomer [18,19]. Presence of PBT in Hytrel was predicted to have good compatibility with Crastin and hence help with the interfacial adhesion of the fibers. Before melt-spinning, Crastin and Hytrel resins are dried in a hot-air resin dryer at 120 °C and 100 °C, respectively.

### 2.2. Bicomponent Co-Extrusion/Spinning

The Crastin and Hytrel resins are dried in a hot-air resin dryer at 120 °C and 100 °C, respectively, for 2 h to maintain the moisture content below 0.04% prior to the melt-spinning process. For the coextrusion process, a filament pilot line from Fibre Extrusion Technology (FET; Leeds, UK) with a side-by-side spinneret die is used. The FET system consists of two melt-pump controlled extruders that feed into a vertical die. The resins are fed into a hopper and melted in separate single-screw extruders with diameters of 20 mm and 25 mm, each equipped with pineapple mixers. The four zones of this extruder are maintained at temperatures of 230 °C, 240 °C, 250 °C, and 260 °C. These two melt streams are adjusted to be discharged at a constant total throughput of 5.57 g/min by melt-pumps (260 °C) into the spin-pack where they merge and are extruded to form a side-by-side bicomponent fiber containing 50% of each melt component. The spinneret which is maintained at 270 °C consists of 12 side-by-side circular profile bores each with a diameter of 0.4 mm. The distance between the spinneret and the take-off rollers is 1.8 m and is the region where hot melt drawing takes place. The take-off rollers are operated at a constant speed of 300 m/min while the godet rolls and final winder where the fibers are collected on a cardboard roll are varied to achieve increasing draw ratios as shown in Table 1 where the draw ratio is defined as take-off roller speed/winder speed. Monocomponent Crastin and Hytrel fibers were also melt-spun as reference samples for characterization studies.

### 2.3. Rheological Characterization

Rheological analysis was performed on a piston-type capillary rheometer Dynisco (Franklin, MA, USA) LCR 7000 for both the resins at high shear rates using a capillary die with a diameter of 0.762 mm and a length to diameter (L/D) ratio of 3. The entrance angle into the capillary was 180°.

### 2.4. Microscopic Analysis

The interfacial morphology of the fibers was studied using an optical microscope (Olympus DSX1000, Olympus, Tokyo, Japan). Fiber samples for observation under the microscope were prepared by two methods, (i) cutting the fibers through their cross-section in a microtome and (ii) casted in epoxy followed by cutting cross-sections and polishing the casted epoxy discs for clarity. Scanning Electron Microscope (SEM) images were taken using JEOL JSM 6390 to observe fiber surface morphology (Tokyo, Japan). The individual fiber samples were placed in a Denton Vacuum (Moorestown, NJ, USA) Desk IV Sputter Coater and palladium was used to coat the samples for 180 s.

### 2.5. Thermal Analysis

Thermal behavior of the polymer pellets, monocomponent fibers and bi-component fibers were investigated using a Differential Scanning Calorimeter (DSC) 2500 (TA Instruments, New Castle, DE, USA) and Tzero Aluminum Hermetic pans (TA Instruments, New Castle, DE, USA) operating in a nitrogen atmosphere with sample weights ranging from 2 to 5 mg. DSC was performed over the temperature range of −50 °C to 250 °C at a heating rate of 10 °C/min and a cooling rate of 5 °C/min. The sample was held isothermally for 2 min after each stage.

Thermo-mechanical analysis of the fibers was performed using a TMA Q400 (TA Instruments) equipped with a film/fiber probe having a gauge length of 8 mm. A force of 1 mN was applied on the single fiber sample to keep the fiber straight and remove any slack. At the beginning of the test, the temperature of the sample was equilibrated at −40 °C and then heated at a rate of 10 °C/min till 80 °C.

### 2.6. Tensile Testing

Tensile characterization on the monocomponent and bicomponent fibers was performed using a KLA T150 UTM having a 0.5 N load cell at room temperature. The samples were attached to a cardboard template with a 30 mm gauge length using double-sided tape. Strain rate of 5 × 10^−2^ s^−1^ was used for the bicomponent fibers to ensure that the fibers broke within 30 s.

### 2.7. Curvature Change Analysis

An established protocol to measure individual fiber curvature within temperature range of 20 °C to 13 °C [16] was used for this study where setup was made to analyze the fiber curvature variation as a function of temperature in a 2-dimensional plane (see Figure S1). A liquid mixture of antifreeze and deionized (DI) water was filled in an aluminum reservoir whose temperature is controlled by a thermoelectric cooler. Fibers of 1 mm in length were cut from the spool and then attached to one end of a glass slide using a fine tipped needle. This glass slide was then attached to the inner wall of the reservoir such that the liquid mixture touched the glass slide and allowed the fiber to float on the liquid surface hence constraining the fiber to the x-y plane. Thermocouples were used to monitor temperature near the fiber. Temperature was varied between the range of −20 °C to 13 °C. The entire setup was installed on an optical microscope and the images of the fibers were taken using camera installed on the microscope. The images were imported to MATLAB script for calculating the fiber curvature by identifying three points on the fiber using Equation (2) [16]. These three points are chosen such that the distance between these points can be measured for all the pictures and *A_tri_* is the area of the triangle formed by the three selected points. It should be noted that these experiments were repeated at least three times for each specimen.

(2) $$C=\frac{1}{\rho }=\frac{4\times A_{tri}}{L_{AB}\times L_{BC}\times L_{AC}}$$

### 2.8. Batting Expansion Test

For expansion testing of non-woven battings cut from the fiber rolls, a macro-expansion probe located in TMA (TA Instruments, New Castle, DE, USA) apparatus was utilized (see Figure S2). Battings of equal thicknesses were obtained and constrained radially. During the test, a force of 0.1 N was applied on the batting sample and the temperature was equilibrated at −40 °C before heating to 80 °C at a rate of 10 °C/min.

## 3. Results

### 3.1. Rheological Behavior

**Figure 1:**
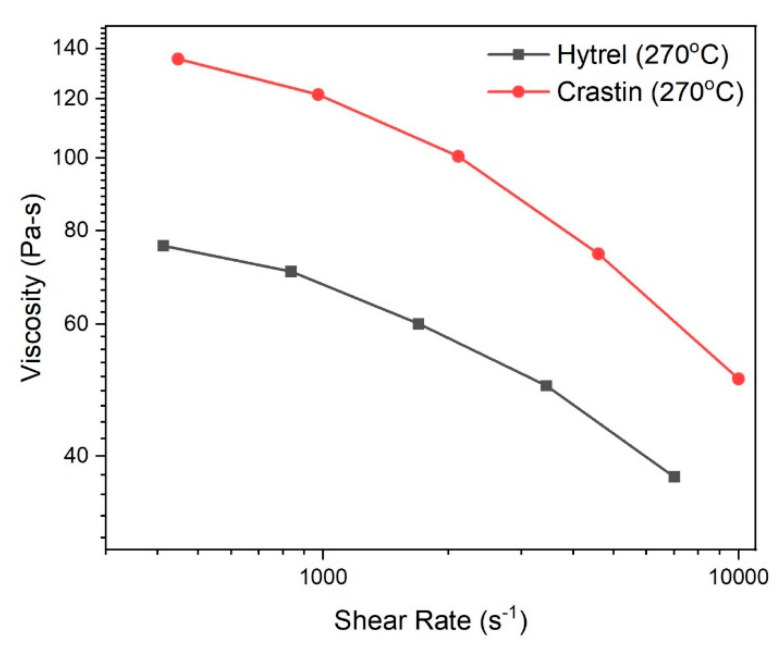
Shear rate vs. Viscosity curves for Hytrel and Crastin.

The viscosity of the melt is affected by the temperature and is an important factor in side-by-side bicomponent fiber spinning since a large mismatch between the two polymer melt viscosities results in melt instabilities at the spinneret surface as the higher viscosity component tries to establish an equilibrium with the pressure inside the spinneret [18]. A discrepancy between the melt viscosities causes the lower viscosity component to encompass the higher viscosity component and a curved interface is observed [19]. For the fibers to exhibit good crimp characteristics, a flat interface at the cross-section is necessary which is only possible with melts of the same viscosity [20]. Figure 1 illustrates the difference in viscosity for Hytrel and Crastin at the processing temperature of 270 °C both of which exhibit typical thermoplastic shear-thinning behavior. Since Hytrel has a lower viscosity than Crastin over the testing range, it is expected that a curved interface would be observed in the fiber cross-section such that it is convex towards Hytrel and, theoretically, leads to a decrease in the crimp potential of the fiber.

The theoretical shear rate at the spinneret can be calculated using the following equation:

(3) $$Ϋ=\frac{4Q}{\pi R^{3}}$$

where Ϋ (s^−1^) is the shear rate, *Q* (m^3^ s^−1^) is the volumetric flow rate, and *R* (m) is the radius of the spinneret bore. For the processing conditions used for the experiments, a shear rate of 1723 s^−1^ is experienced by the polymer melts. A viscosity difference of 45.5 Pa-s is exhibited by the melts at this shear rate as seen in Figure 1 and is the reason for the interfacial curvature occurring in the fiber cross sections.

### 3.2. Fiber Processing and Morphology Optimization

Initially, the extrusion temperature for Crastin and Hytrel were set at 260 °C, and the melt throughput was set at 1 g/min from each hole; however, the fibers then exhibited a curved interface of Crastin towards Hytrel. Moreover, the fibers could not be drawn more than 1.5 without delamination. (see Figure S3a) for the image of the fiber cross section). It was also evident that while the ratio between Hytrel and Crastin could be controlled, i.e., 25% vs. 75%, 50% vs. 50%, and 75% vs. 25% (see Figure S3b), a previous curvature prediction [15] suggests symmetrical cross-section with a straight interface in the center for the greatest crimp potential.

**Figure 2:**
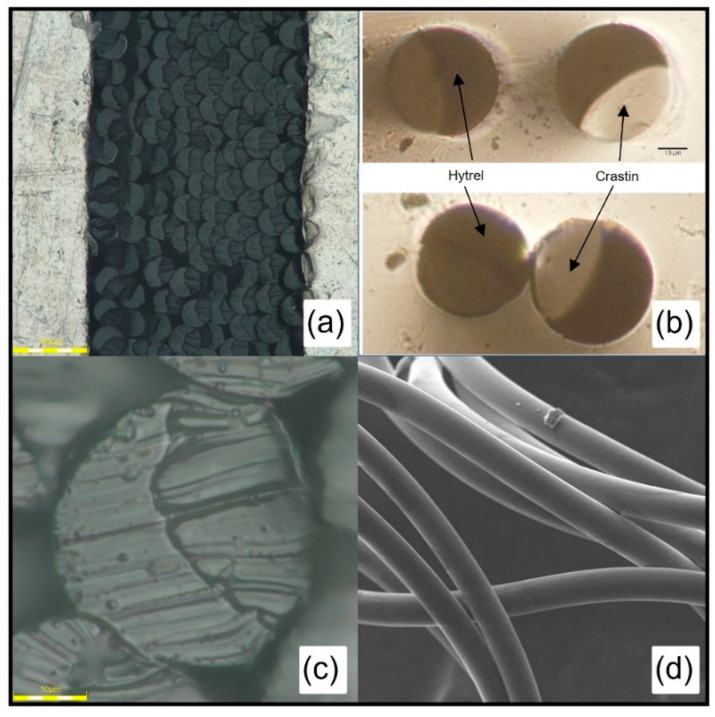
Side-by-side fiber cross sections and surface morphology. (**a**) displays overall filament morphology, (**b**) shows zoomed in photo showing two distinct phases, (**c**) displays a zoome in photo of single filament, and (**d**) SEM of the fiber surface.

To reduce the interfacial curvature in the cross-section, the melt throughput was reduced to 0.5 g/min through each hole which reduced shear experienced by the melt which helped reduce the amount of die swell after exiting the spinneret. The spin pack temperature was also increased from 260 °C to 270 °C to reduce the viscosity and aid in interfacial curvature reduction. As seen in Figure 2a–c, fiber cross sections show Hytrel and Crastin side-by-side domains with Crastin encompassing Hytrel due to its lower viscosity in the melt state; the interfacial curvature is less pronounced than those spun at 260 °C at 1 g/min production. In addition, no doglegging was encountered during the melt-spinning process while stable drawing of fibers up to 3.67 draw ratio was observed without delamination. SEM images of the fibers in Figure 2d show a smooth morphology with no surface imperfections or beading effect. During processing, drawing of the fibers takes place between the take-off rolls and the winder as the draw ratio is varied for each trial which leads to strain-induced crystallization in the fibers. Diameter of the fibers decreases as the drawing on the fibers is increased (see Figure S4); diameters ranged from 49 µm to 33 µm for draw ratio of 1.67 to 3.67. Since the take-off speed is constant for the all the trials, the crystallization rate for drawing between the spinneret and take-off rolls stays the same [21].

### 3.3. Thermal Analysis

**Figure 3:**
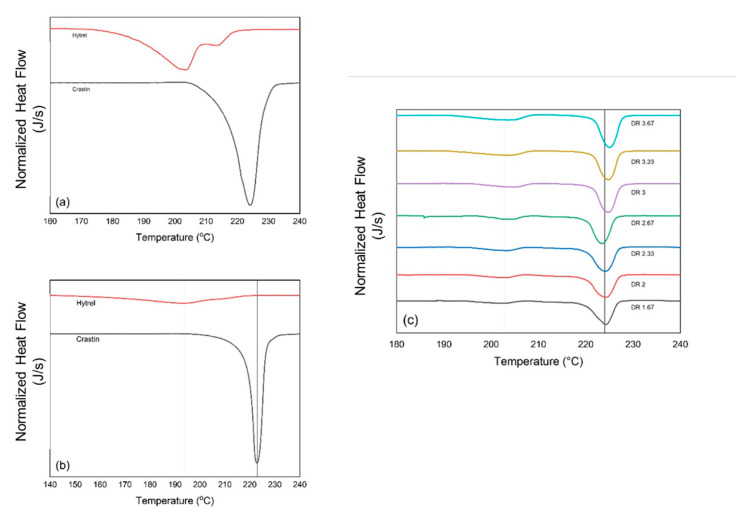
DSC curves for (**a**) Hytrel and Crastin pellets, (**b**) Hytrel and Crastin monofilament fibers, and (**c**) Bi-component fibers with respective draw ratios (DR).

DSC curves in Figure 3a,b for Crastin show a sharp melting peak for both the pellet and fiber although at slightly different temperatures of 223.98 °C and 222.93 °C, respectively, although for Hytrel the melting peaks are broader and less pronounced for both pellet and fiber at 203.52 °C and 193.23 °C, respectively. It can also be observed that the split peak visible for Hytrel pellet disappears in the curve for Hytrel monocomponent fiber.

DSC curves for the bicomponent fibers in Figure 3c show that the melting peak for draw ratios 1.67, 2, 2.33, and 2.67 is close to 223 °C, but the melting peak shifts to higher temperatures of around 225 °C for fibers with draw ratios of 3, 3.33, and 3.67. It can also be observed that the broadness of the peaks decreases with increasing draw ratio. It can be postulated that due to the low throughput of 0.47 g/min per hole, the fibers formed just after the spinneret have a larger surface area per unit mass due to lower diameter and hence lead to a higher rate of crystallization in the fiber [21]. Due to this, all the bicomponent fibers are partially crystalline for all draw ratios as a direct result of strain-induced crystallization of the molecular chains; some of this evidence is noted in Table 2. A reason for the absence of cold crystallization for all the trials could be that the PBT helical structure restricts the melt to transform into an amorphous state upon cooling and instead prefers a crystalline state [1]. As a result, the degree of crystallinity is saturated and further drawing can only slightly affect the crystallite size [22]. For the Hytrel melting peaks, similar peak shifting behavior is observed along with increase in the peak height.

### 3.4. Tensile Properties

**Figure 4:**
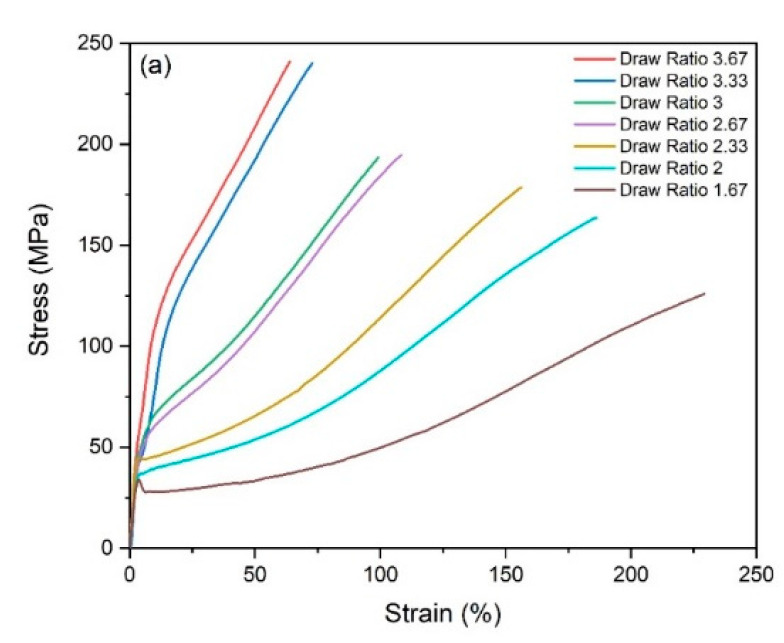

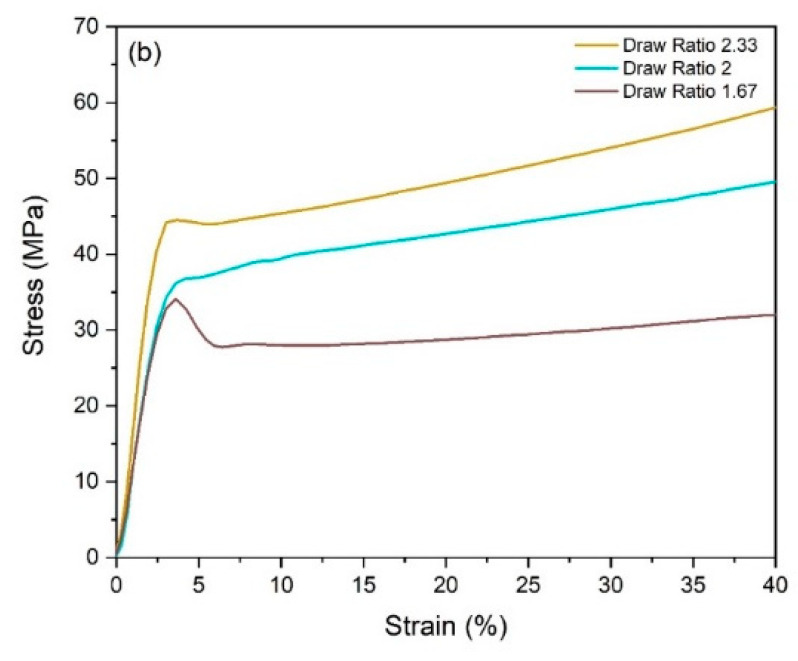
Tensile curves for the PBT bicomponent fibers. (**a**) shows all representative curves with different DR, while (**b**) shows zoomed in section (strain 0–40) of DR = 1.67, 2.0, and 2.33.

The effect of strain-induced orientation on the bicomponent fiber mechanical properties can be seen in Figure 4. The fibers were never split down the interface during tensile testing, which confirms stable interfacial adhesion of the two polymer components. With an increasing draw, strain to break for the fibers is reduced, while the corresponding stress and initial modulus values increase. For the lower draw ratios of 1.67, 2, and 2.33, yield points and subsequent elongation are clearly seen which indicate the presence of undrawn amorphous polymer chains in the fibers. The rigid Crastin chains dominate tensile behavior as observed in the initial region up to 8–10% strain in the lower draw ratios of 1.67, 2 and 2.33 (Figure 4b); when draw ratio is higher than 2.33, no clear yield point is observed which may be due to increased molecular orientation. Only at the lowest draw ratio of 1.67 can yielding be observed followed by a small plateau region which could correspond to the formation of metastable stress-induced β phase observed in PBT around 4–12% strain [23].

**Figure 5:**
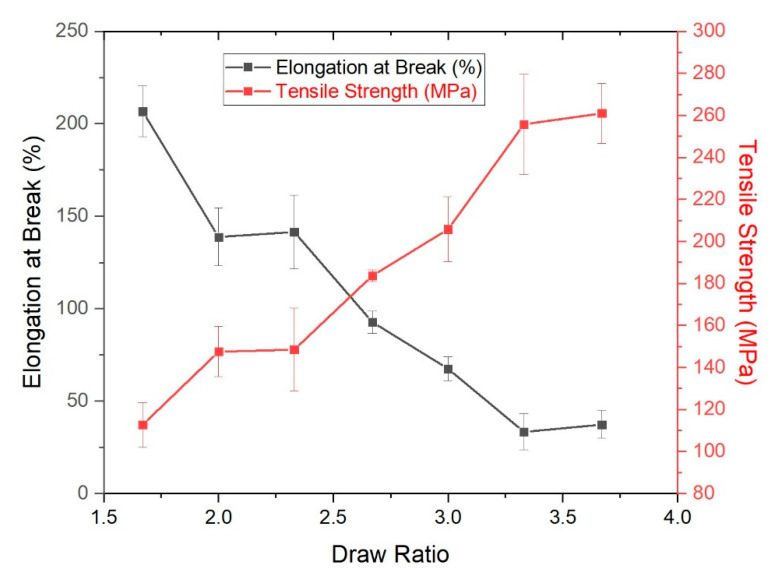
Elongation at Break and Tensile Strength of Bicomponent Fibers as a function of Draw Ratio.

Figure 5 confirms the expected result of increasing the draw ratio which decreases the elongation at break from 206.7% to 37.4% since the molecular chains are increasingly oriented. Further stress causes the remaining unoriented chains to stretch and eventually break which also results in a higher tensile strength of 260.9 MPa for the highest draw ratio while a tensile strength of 112.7 MPa is seen for the fiber with a lower draw ratio. The fibers initially go through yielding followed by a plateau region of increasing strain with no increase in stress followed by deformation characterized by the elastomeric component, Hytrel before eventually breaking. Fibers with the draw ratio of 2.33 have a good balance of higher elongation at break suggesting that the Hytrel chains are predominantly in an amorphous state while the presence of the sharp yielding suggests that Crastin sustains the initial loading and shows increased chain orientation as compared to draw ratios of 1.67 and 2. Table 3 shows the calculated Young’s Modulus for the fibers and an increasing trend is observed which is in line with the effect of drawing. This trend is also consistent with the increase of crystallinities with draw ratio in Table 2. All the values are higher than bulk Hytrel’s modulus of 0.19 GPa and lower than that of Crastin at 2.6 GPa; it is notable that the fiber moduli of Hytrel and Crastin are very similar to bulk values, which are 0.18 GPa and 2.4 GPa, respectively. In Equation (1), the curvature is inversely proportional to $\left(\frac{\pi^{2}}{8}-\frac{8}{9}\right)+\left(\frac{\pi^{2}}{16}-\frac{4}{9}\right)n+\left(\frac{\pi^{2}}{16}-\frac{4}{9}\right)\frac{1}{n}\;$. One may conjecture that moduli differences of Hytrel and Crastin components becomes larger with higher draw ratio owing to higher crystallinity of Crastin with DR (Table 2). Unfortunately, it is difficult to measure the moduli of each component of a conjoined bicomopnent fiber as used in the current study and cannot be confirmed.

### 3.5. Thermo-Mechanical Properties

**Figure 6:**
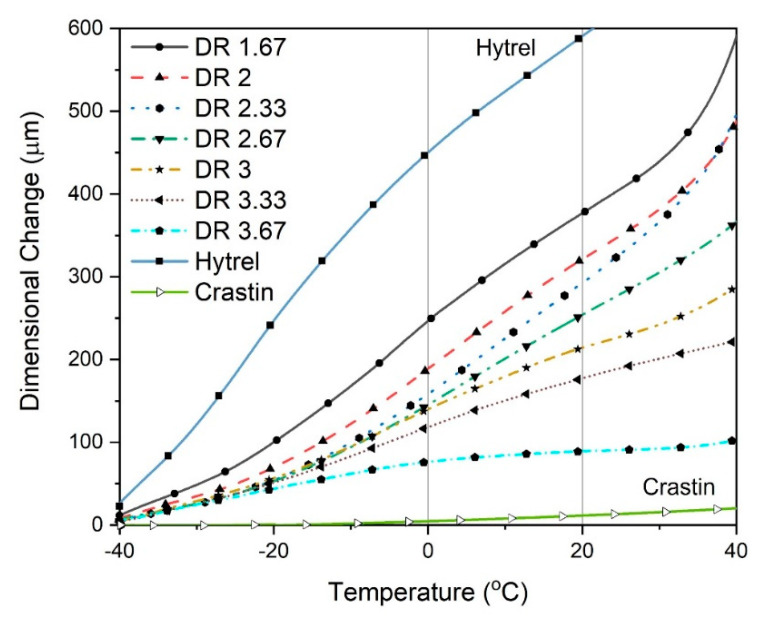
TMA curves for Monocomponent fibers (labelled Hytrel and Crastin) and Bicomponent fibers with respective draw ratios (DR).

To understand the effect of draw ratio (and consequently the fiber diameter) on dimension change its CTE, TMA tests were performed on each individual fibers in the temperature range of −40 °C to 40 °C. As seen in Figure 6, a minimal dimensional change is observed for the monocomponent Crastin fiber as compared to the monocomponent Hytrel fiber which shows the largest dimensional change. These two curves are effectively the lower and upper bounds for the bicomponent fiber dimensional change observed. Hytrel shows a tendency to expand continuously above its glass transition temperature of −25 °C; also observed is a characteristic thermal shrinkage hump around 10 °C that could be associated to the oriented amorphous chains which undergo chain coiling as the temperature increases. The Crastin monocomponent fiber, on the other hand, is partially crystalline due to melt-spinning and hence shows very low dimensional change in the test range below its glass transition temperature of 55 °C.

**Figure 7:**
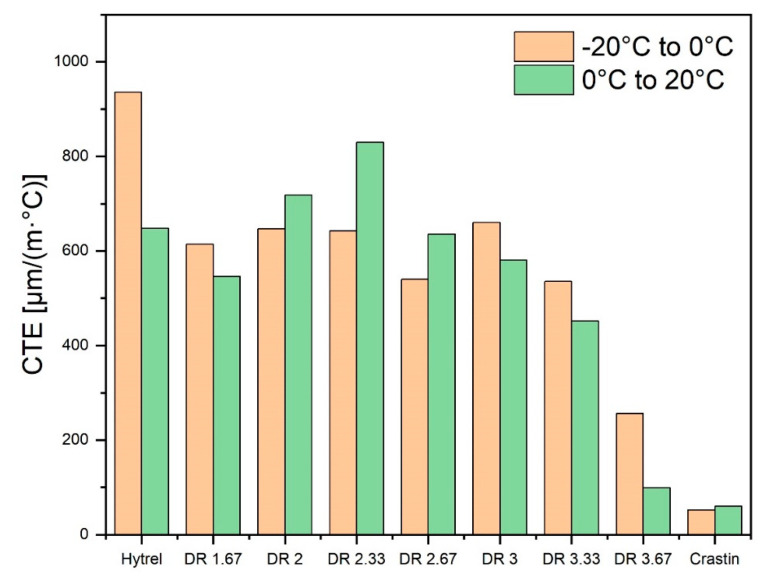
CTE vs. Draw ratios of bicomponent (2nd to 8th columns) and monocomponent fibers (Hytrel and Crastin, 1st and last columns) in the temperature ranges of −20 °C to 0 °C and 0 °C to 20 °C.

For the bicomponent fibers, a trend of increasing linear expansion with reduction in draw ratio is observed. These curves still show the characteristic hump near 10 °C due to the Hytrel component. As the draw ratio increases, the molecular orientation of the chains increases and dimensional change is restricted due the increased crystalline structure while for lower draw ratios, the presence of more amorphous region allows for larger gradual extension. At first glance, the trend seems to contradict prediction from Equation (1) as higher magnitude of thermomechnical change is expected with higher DR due to (i) smaller diameter, and (ii) larger mismatch of moduli. One should note that Figure 6 shows the linear dimensional change in uniaxial tensile direction, not a fiber curvature described by Equation (1); this distinction will be further explained when CTE values are compared in Figure 7. Based on Figure 6, we conjecture the mechanism behind fiber curvature as following: Hytrel tends to linearly expand while the Crastin component limits the linear expansion, thus the net effect is fiber to curl with response to temperature. Bicomponent fibers with 75% Hytrel/25% Crastin and 25% Hytrel/75% Crastin were also tested; however, the dimensional change obtained was significantly less than for 50% Hytrel, 50% Crastin fibers (see Figure S5); this result reminds the importance of the symmetrical interface along the center as predicted [14].

CTE values are plotted in Figure 7 for bicomponent and monocomponent fibers based on Figure 6 between the temperature ranges of −20 °C to 0 °C and 0 °C to 20 °C. The CTE values of Crastin from Figure 7 are similar to its bulk value of 144 µm °C^−1^ m^−1^. Hytrel values, on the other hand, are 3 to 4 times greater than the bulk values of 200 µm °C^−1^ m^−1^, as Hytrel is above its T_g_ of −25 °C and chains are able to move freely under the small load on the fiber during testing. The CTE for Hytrel monocomponent fiber increases between −20 °C and 0 °C and decreases between 0 °C to 20 °C while Crastin shows the lowest CTE as temperature is still below its glass transition temperature of 55 °C. Moderately drawn fibers of draw ratio 2.33 show the highest CTE when compared to lower and higher drawn fibers. The discrepancy could be explained by the increasing modulus of the Crastin component which might be hindering the overall fiber expansion after a draw ratio of 2.33 whereas below it, a lower modulus could be allowing to greater thermal shrinkage resulting in a lower CTE value. In Equation (1), the magnitude of bicomponent CTE is proportional to the fiber curvature; as such, one may expect the highest fiber curvature at DR of 2.33.

### 3.6. Fiber Curvature Analysis

**Figure 8:**
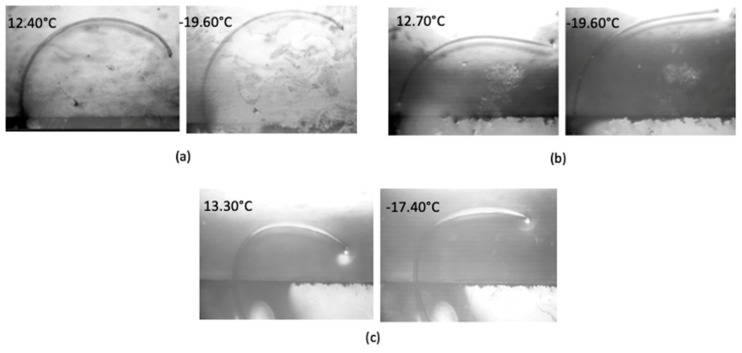
Optical images of fiber curvature change at different temperatures. (**a**–**c**) display three different fiber samples with repeatable curvature change within the temperature range.

Figure 8 Based on the tensile and thermomechanical tests, fibers with draw ratios of 2.33 were selected for curvature analysis tests as shown in Figure 8. It was observed that at the starting temperature of around 13 °C, the initial fiber curvature is higher and as the temperature reduces to near −20 °C, the curvature of the fiber decreases in the constrained 2D plane of the water surface. An average curvature change of 0.12 mm^−1^ was observed for the fibers for a temperature range of 32 °C. To illustrate how this compares to a predicted curvature value, Table 4 shows predicted curvature values at different fiber diameter based on bulk CTE and moduli of Hytrel and Crastin.

The fiber diameters in Figure 8 are approximately 41 µm; the average curvature change of 0.12 mm^−1^ is about five times greater than the predicted value (40 µm diameter, ΔT = 30 °C) in Table 4 based on bulk material properties. This difference may stem from (i) bicomponent fiber CTE being significantly larger (Figure 7), and (ii) moduli ratio being different from bulk values.

**Figure 9:**
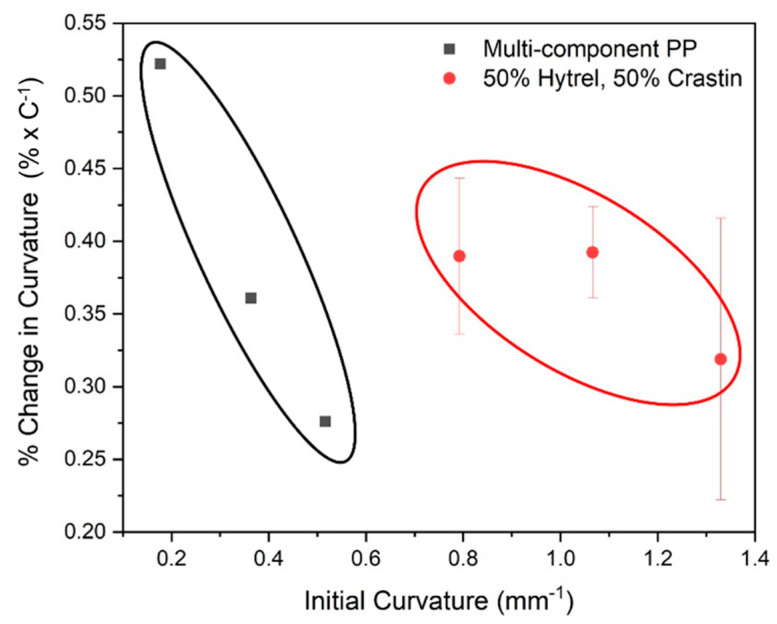
Percent change in curvature with respect to temperature vs. initial fiber curvature for multi-component polypropylene fibers and bicomponent PBT fibers.

Figure 9 shows a plot of the % change in the fiber curvature over the temperature range as a function of the initial curvature of the fibers. The data for Hytrel–Crastin bicomponent fibers was compared with data available for triangular cross-section multi-component (PP) fibers made of isotactic PP and syndiotactic PP with a random ethylene propylene copolymer (co-EP) as the middle interfacial layer [15]. These fibers had an edge length of approximately 50 μm and were drawn to a ratio of 2.51, compared to the bicomponent fibers from present study, which had a diameter of approximately 40 μm and drawn to a ratio of 2.33. Comparing the two fibers, initial curvature for the PP fibers was lower which indicated lower crimping of the fibers as compared to the Hytrel–Crastin bicomponent fibers in the present study. The PBT bicomponent fibers, in general, show comparable change in curvature as for the PP fibers [15]. These results reflect how the curvature of the fiber is dependent on stress distribution in the fiber which is affected by drawing, fiber orientation, and the interfacial curvature. When compared to bulk properties, fibers show increase in the CTE and the modulus which explains why the curvature change is higher than predicted in Table 4, which assumes no initial stresses and no initial curvature in the fibers.

### 3.7. Batting Expansion Testing

**Figure 10:**
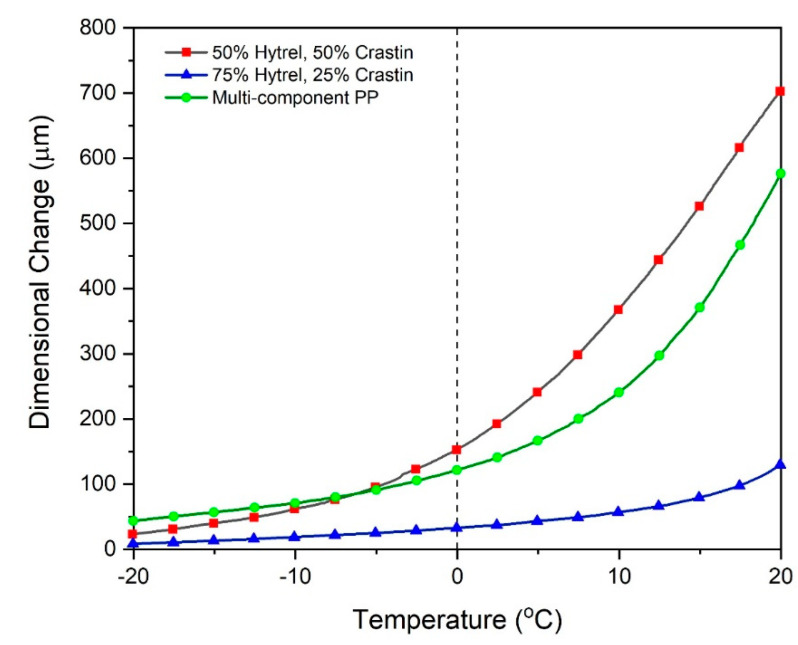
Batting expansion test for bicomponent fibers with compositions 50% Hytrel and 50% Crastin, 75% Hytrel and 25% Crastin, multi-component PP fibers between −20 °C to 20 °C.

PP fibers with a triangular cross section drawn to a ratio of 2.5 and having an edge length of 50 μm were obtained as used by DeCristofano et al. [15]. The data for expansion of these fibers along with bicomponent fibers is plotted in Supporting Figure S6 which shows the highest dimensional change of 1295 μm for the PP fibers while 50% Hytrel and 50% Crastin fibers show a maximum change of 890.3 μm followed by 75% Hytrel and 25% Crastin with 458.2 μm in the −40 °C to 40 °C temperature range. If the battings are expected to be functionally used in the range of −20 °C to 20 °C, as observed in Figure 10, bicomponent fibers with 50% Hytrel and 50% Crastin show the highest change of 704 μm followed by PP fibers with 563 μm change and then 134 μm for the 75% Hytrel and 25% Crastin fibers.

The low dimensional change between −20 °C and 0 °C is due to the thermal shrinkage experienced by the individual fibers as seen in Figure 6. Above 0 °C, a greater dimensional change is experienced, which is accelerated by the temperature increase that provides mobility for the chains to relax and the fibers tend to curl further while sightly increasing in its length. As seen in Figure 9, the fiber curvature increases at a higher temperature and this change is compounded in the batting causing the fibers to push on each other and expand the batting.

## 4. Conclusions

Melt-spinning of side-by-side bicomponent fibers with a low modulus, high CTE component (Hytrel) and a high modulus, low CTE component (Crastin) was studied for their thermoresponsive properties for potential application in battings for dynamic thermal insulation. These fibers showed excellent interfacial stability at all draw ratios which could be tailored based on the amount of draw which imparts the mechanical and thermal properties. Tensile tests indicated that drawing led to orientation of the chains and the fibers lost their characteristic elastomeric properties stemming from the Hytrel component as the amorphous regions are oriented due to straining; this was corroborated by the lower CTE of 395.5 μm/(m.°C) for the highest draw ratio as compared to 606.25 μm/(m.°C) for the lowest draw ratio seen during TMA testing. As expected, the PBT crystallinity increased from 13.49% for the lowest drawn fiber to 17.7% for the highest draw ratio and the melting peaks shift slightly to the right indicating larger crystallite size. Based on these results, fibers with draw ratio of 2.33 yielded a balance of mechanical (tensile with yield) and thermal (highest CTE) properties and exhibited a notable fiber curvature. The curvature results show that the current PBT bicomponent fibers showed a larger initial curvature and similar percent change in curvature as compared to similarly fabricated PP multi-component fibers [15]. Non-woven battings from the current study showed a 12.8% expansion from an initial thickness of 5.5 mm in the temperature range of −20 °C to 20 °C, comparable or better than battings made from PP-based fibers. Such result is promising for the application of these fibers for thermal insulation as the curvature change would affect the crimping of the fibers and result in batting thickness increase or decrease based on the ambient temperature.

## Figures and Tables

**Table 1:** Fiber drawing parameters.

Take-Off (m/min)	Godet Roll 1 (m/min)	Godet Roll 2 (m/min)	Godet Roll 3 (m/min)	Winder (m/min)	Draw Ratio
300	325	350	400	500	1.67
300	400	450	500	600	2.00
300	500	550	600	700	2.33
300	500	600	700	800	2.67
300	600	700	800	900	3.00
300	600	750	900	1000	3.33
300	700	850	1000	1100	3.67

**Table 2:** Enthalpies for Hytrel and Crastin Components in Bicomponent Fibers.

Draw Ratio	Hytrel Enthalpy (J/g)	Crastin Enthalpy (J/g)	Crastin Crystallinity (%)
1.67	4.592	19.625	13.488
2.00	5.976	20.475	14.072
2.33	7.114	21.042	14.462
2.67	5.996	21.124	14.518
3.00	7.814	23.587	16.211
3.33	7.959	22.278	15.311
3.67	9.682	25.759	17.704

**Table 3:** Draw Ratio vs. Young’s Modulus.

Draw Ratio	Young’s Modulus (GPa)
1.67	1.168 ± 0.012
2.00	1.472 ± 0.125
2.33	1.754 ± 0.130
2.67	1.798 ± 0.065
3.00	1.906 ± 0.120
3.33	2.001 ± 0.145
3.67	2.165 ± 0.187

**Table 4:** Predicted Curvature change based on bulk properties as a function of temperature change and fiber diameter.

**Temperature Difference (°C)**	**Diameter (μm)**		
	40	30	20
		**Curvature (mm^−1^)**	
40	0.032	0.043	0.065

## Data Availability

The data presented in this study are available on request from the corresponding author.

## Supplementary Materials

The following supporting information can be downloaded at: https://www.mdpi.com/article/10.3390/polym14142757/s1, Figure S1: Fiber Curvature Change (a) Schematic and (b) Experimental Setup; Figure S2: Thermal expansion module in TMA; Figure S3: Delamination at Interface for Fiber Spun at 260 °C; Figure S4: Effect of Drawing on Fiber Diameter; Figure S5: TMA Curves for Fibers with varying Hytrel Composition; Figure S6: Batting expansion test for bicomponent fibers with compositions 50% Hytrel and 50% Crastin, 75% Hytrel and 25% Crastin, multi-component PP fibers between temperature range of −40 °C to 80 °C.
