# Supplementary material for: Temperature Responsive PBT Bicomponent Fibers for Dynamic Thermal Insulation
Source: Polymers (Basel). 2022 Jul 6;14(14):2757. doi: 10.3390/polym14142757 (PMC9323749; doi:10.3390/polym14142757)
Supplement: Supplementary file 1 [file polymers-14-02757-s001.zip › polymers-1793280-supplementary.pdf]

Temperature Responsive PBT Bicomponent Fibers for Dynamic Thermal Insulation

Ninad Khadse [1], Rebecca Ruckdashel [1], Shnaidie Macajoux [1], Hongwei Sun [2], Jay Hoon Park [1,*]

[1] Department of Plastics Engineering, University of Massachusetts Lowell, Lowell, MA 01854, USA

[2] Department of Mechanical and Industrial Engineering, Northeastern University, Boston, MA 02115, USA

Supporting Document

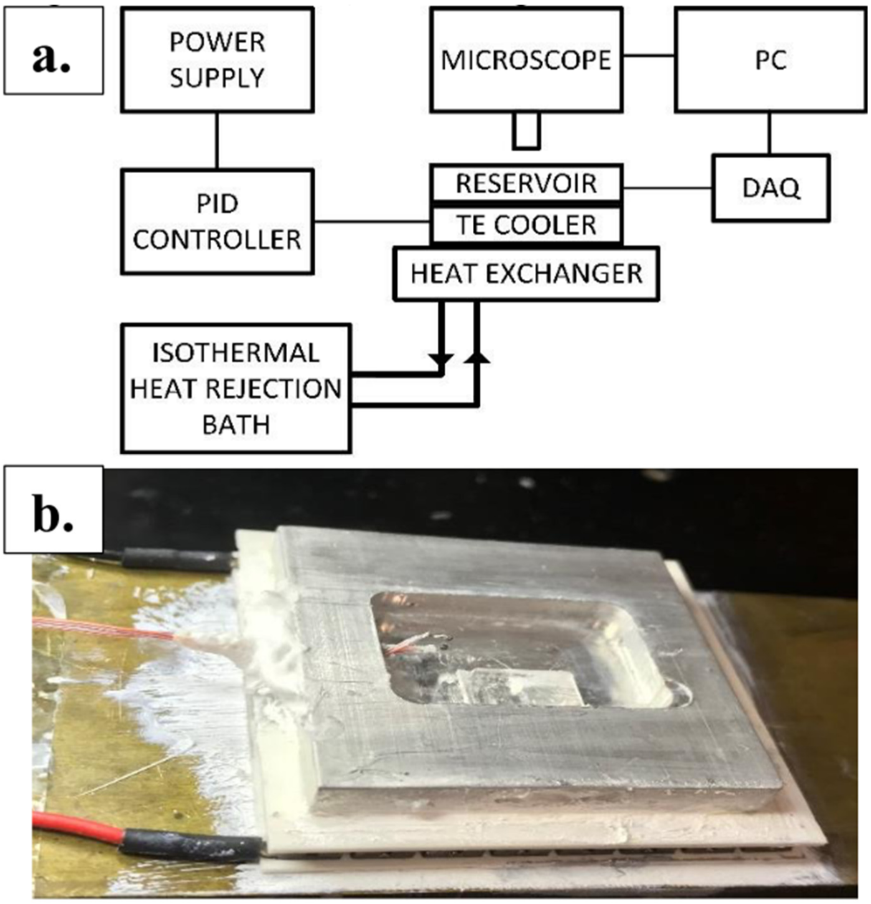

Figure S1. *Fiber Curvature Change (a) Schematic and (b) Experimental Setup*

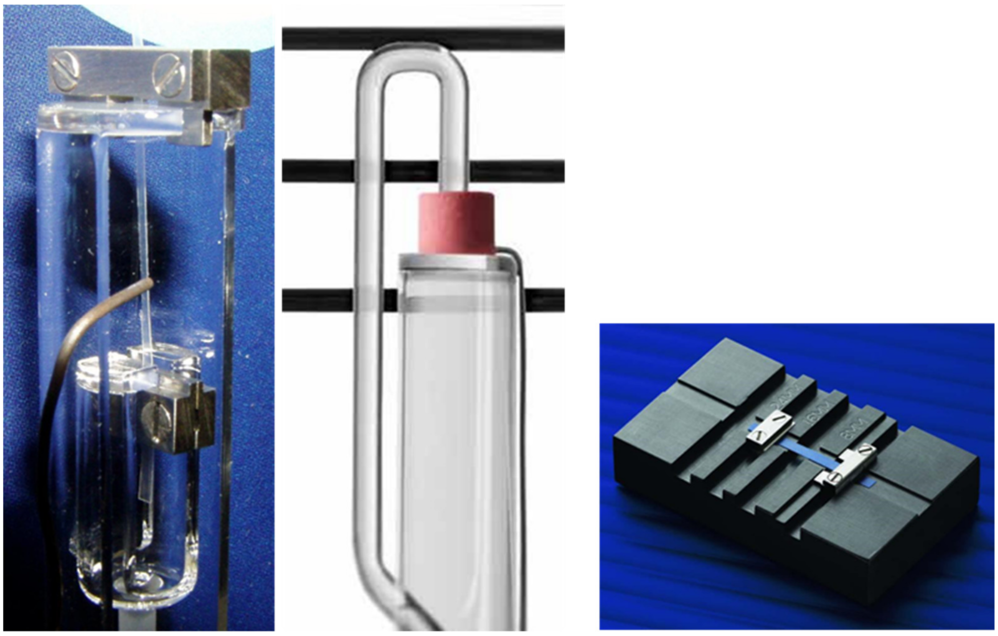

Figure S2. A) Left shows probe used to contain non-woven battings to measure thermally-induced expansion in the axial direction, while b) right shows fiber probe used to measure linear expansion (in fiber axis) with respect to temperature.

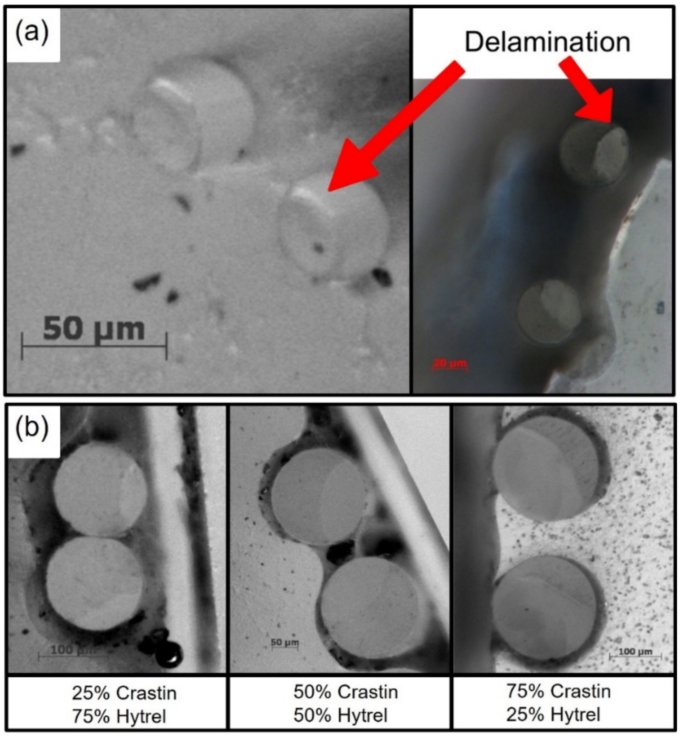

Figure S3. *Delamination at Interface for Fiber Spun at 260C*

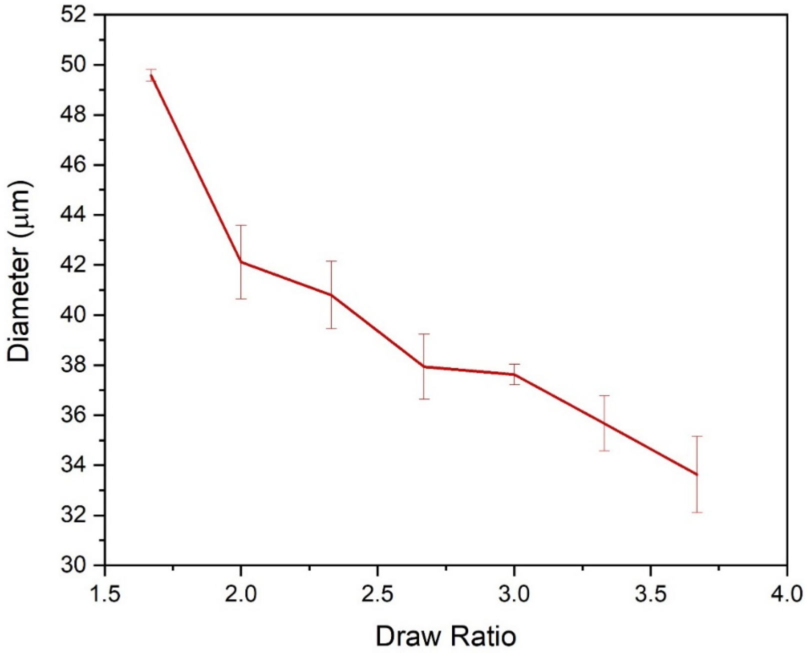

Figure S4. Effect of Drawing on Fiber Diameter

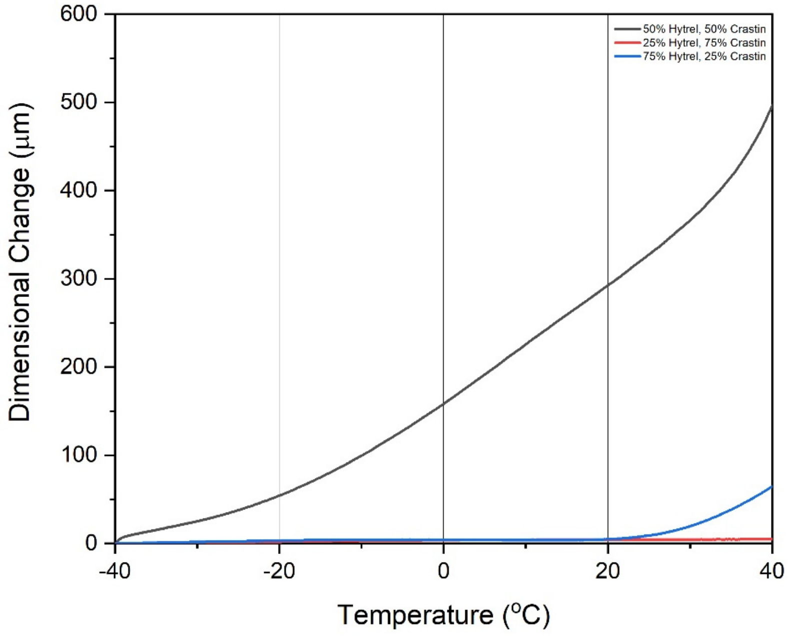

Figure S5. TMA Curves for Fibers with varying Hytrel Compositions

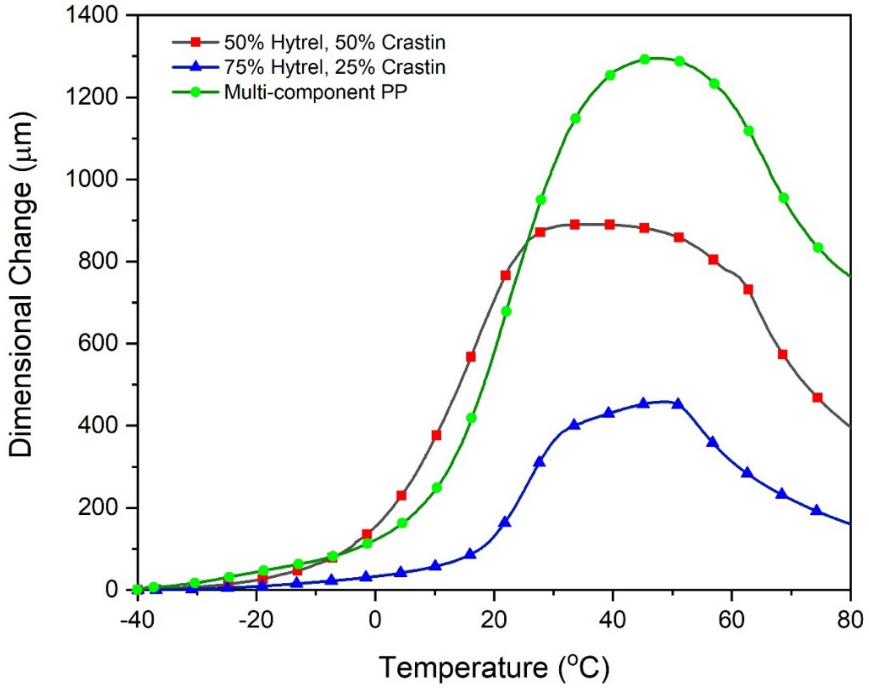

Figure S6. Batting expansion test for bicomponent fibers with compositions 50% Hytrel and 50% Crastin, 75% Hytrel and 25% Crastin, multi-component PP fibers between temperature range of -40°C to 80°C
